# Myanmar Dengue Outbreak Associated with Displacement of Serotypes 2, 3, and 4 by Dengue 1

**DOI:** 10.3201/eid1004.030216

**Published:** 2004-04

**Authors:** Hlaing Myat Thu, Kym Lowry, Thein Thein Myint, Than Nu Shwe, Aye Maung Han, Kyu Kyu Khin, Kyaw Zin Thant, Soe Thein, John Aaskov

**Affiliations:** * Queensland University of Technology, Brisbane, Australia; † Department of Medical Research, Yangon, Myanmar; ‡ Yangon Children’s Hospital, Yangon, Myanmar; § Mawlamyaing General Hospital, Mawlamyaing, Myanmar

**Keywords:** Dengue virus, dengue hemorrhagic fever, Myanmar, dengue phylogeny

## Abstract

In 2001, Myanmar (Burma) had its largest outbreak of dengue—15,361 reported cases of dengue hemorrhagic fever/dengue shock syndrome (DHF/DSS), including 192 deaths. That year, 95% of dengue viruses isolated from patients were serotype 1 viruses belonging to two lineages that had diverged from an earlier, now extinct, lineage sometime before 1998. The ratio of DHF to DSS cases in 2001 was not significantly different from that in 2000, when 1,816 cases of DHF/DSS were reported and dengue 1 also was the most frequently isolated serotype. However, the 2001 ratio was significantly higher than that in 1998 (also an outbreak year) and in 1999, when all four serotypes were detected and serotypes 1, 2, and 3 were recovered in similar numbers. The large number of clinical cases in 2001 may have been due, in part, to a preponderance of infections with dengue 1 viruses.

Dengue is a disease caused by four serotypes of a flavivirus of the same name (*1*). Infection with these viruses may be inapparent, or it may result in disease varying in severity from a mild influenza-like illness to hemorrhagic fever and hypovolemic shock, which may be fatal if untreated (*1*). Dengue is an important source of illness and death in tropical nations, particularly in Southeast Asia and Central and South America (*2*). In 1998, a pandemic of dengue resulted in 1.2 million cases of dengue hemorrhagic fever (DHF) in 56 countries (*3*). In many countries in Asia where this disease is endemic, outbreaks occur in cycles of 3 to 5 years due, perhaps, to enhanced infection with one serotype caused by cross-reactive antibody produced in response to an earlier infection with a second serotype (*4*), rather than to climatic effects (*5*). Furthermore, the incidence of disease and its severity vary between primary and secondary infections and between infections with different dengue virus serotypes (*6*–*8*). All four dengue virus serotypes circulate in countries in Southeast Asia from Myanmar to Indonesia (*9*–*13*), and no outbreaks caused by single virus serotypes, as have been seen in dengue non-endemic areas such as Cuba or Australia (*14*,*15*), have recently been reported. Phylogenetic studies have shown regular extinction of strains of dengue viruses in single locations and emergence of new strains (*16*,*17*), and it has been suggested that the appearance of more fit or more pathogenic viruses may occur as a result of immunologic selection during periods of intense transmission during outbreaks (*18*). While weak selective pressure on the envelope (E) protein gene of some dengue viruses is evident (*19*), the most extensive changes in virus genotypes appear to be due to recombination or to possible genetic bottlenecks (*16*,*17*,*20*). Given these observations, and the finite pool of hosts in most locations (humans and selected species of *Aedes* mosquitoes), perhaps it is surprising that greater competition between the four serologically related serotypes of dengue virus has not been observed, e.g., the complete exclusion of two or three serotypes from an ecologic niche.

## Patients and Methods

### Serology

Acute- and, where possible, convalescent-phase serum samples were obtained from patients admitted to the Yangon Children’s Hospital with a clinical diagnosis of DHF (*1*). A patient with a confirmed case of dengue fever was one who met any of the following criteria: 1) paired sera showed a fourfold or greater rise in hemagglutination inhibiting (HI) antibody titer against dengue virus (*21*); 2) a convalescent-phase serum sample produced an immunoglobulin (Ig) G reaction (titer equivalent to an anti-dengue virus HI titer of >2,560), an IgM reaction in a commercial “rapid” dengue test (*22*), or both; or 3) dengue virus was recovered by culturing 140 μL of acute-phase serum on 25-cm^2^ monolayers of C6–36 *A. albopictus* cells for 7 days. The serotype of the virus was determined by performing indirect immunofluorescence (*23*) on cells from the cultures of C6–36 cells used for virus isolation with flavivirus-, dengue-, or serotype-specific monoclonal antibodies (*23*,*24*).

### Phylogenetic Analyses

RNA was extracted from virus isolates with a commercial kit (Qiagen, Hilden, Germany) according to the manufacturer’s instructions, transcribed to cDNA, and amplified by polymerase chain reaction as described previously (*24*). The cDNA was purified and then sequenced on an automated sequencer (Applied Biosystems, Inc., Foster City, CA) (*24*). Nucleotide sequences were edited, compared, and analyzed with software (EclustalW, Ednapars, Ednadist, Ekitsch) from the Australian Genome Information Service (available from: http://www.angis.org.au). Bootstrap values were derived from 100 re-samplings using software Eseqboot/Ednadist/Eneighbour/Consense also from the Australian Genome Information Service. Additional nucleotide sequences used in the phylogenetic analyses are listed as country, year of isolation, dengue virus serotype, and GenBank accession number, i.e., (Sin[gapore]90D1, M87512; Abid[jan]99D1, AF298807; Mal[aysia]72D1-12, AF231721; Thai64D1, AF180817; Thai58D1, D10513; Phil[ippines]D1, D00503; Nauru74 D1, U88535; Mex[ico]83D1, D00504; Jam[aica]77D1, D00501; Japan43D1M, AB074760; Haw[aii]44D1, X76219; Lao96D1, AB003090; China80D1, AF350498; Camb[odia]98D1, AF309641).

## Results

Dengue is endemic in Myanmar. Outbreaks have occurred in 3- to 5-year cycles of increasing magnitude since the first recorded outbreak in the country in 1970 (Table 1). The outbreak in 2001 (15,361 cases of DHF/dengue shock syndrome [DSS]) was the largest on record.

**Table 1:** Annual dengue hemorrhagic fever cases, Myanmar

Y	Cases	Y	Cases	Y	Cases	Y	Cases
1970	1,654	1978	2,029	1986	2,114	1994	11,647
1971	691	1979	4,685	1987	7,331	1995	2,218
1972	1,013	1980	2,026	1988	1,178	1996	1,854
1973	349	1981	1,524	1989	1,196	1997	4,006
1974	2,477	1982	1,706	1990	6,318	1998	12,918
1975	6,750	1983	2,756	1991	6,770	1999	5,753
1976	3,153	1984	2,273	1992	1,685	2000	1,816
1977	5,364	1985	2,666	1993	1,979	2001	15,361

In 1998 and 1999, all four dengue virus serotypes (DENV-1–4) were recovered from patients in the Yangon Children’s Hospital; DENV-1, -2, and -3 were recovered in approximately similar ratios each year (Table 2). In 2000 and 2001, no DENV-4 was recovered, and DENV-1 was recovered more frequently than any other serotype. In 2001, 95% of isolates were DENV-1. No significant difference (p > 0.05, chi-square test) was found in the rate of isolation of dengue viruses from seronegative serum samples for each of these years.

**Table 2:** Dengue virus (DENV) serotypes recovered from patients in Yangon Children’s Hospital, 1998–2001

	1998	1999	2000	2001
Clinical cases	4,596	2,342	1,100	5,616
Paired sera	709	739	501	1870
Confirmed cases	496	373	274	1,481
Sera with HI titer <80 for virus isolation	54	30	42	236
DENV-1 isolates	9	2	6	115
DENV-2 isolates	5	5	1	1
DENV-3 isolates	9	3	1	3
DENV-4 isolates	1	1	0	0
Mixed virus isolates	0	0	0	2 (dengue 1+2)

Accompanying the change in the relative proportions of dengue virus serotypes recovered from patients in the Yangon Children’s Hospital was a significant change in the relative proportion of clinically diagnosed DHF and DSS cases (1998: 3,194 DHF, 1,402 DSS; 1999: 1,741 DHF 601, DSS; 2000: 896 DHF, 224 DSS; 2001: 4,511 DHF, 1,105 DSS), i.e., DSS occurred in a smaller proportion of patients in 2000 and 2001 than in 1999 or 1998 (p < 0.01, chi-square test). Hemorrhagic signs and symptoms developed in most of the dengue fever patients in 2001; such patients were distinguished from DHF patients only on the basis that their platelet levels were >100,000/mm^3^.

Of the patients with a laboratory-confirmed dengue infection and sufficient clinical and laboratory detail to confirm the grade of infection (990 patients), almost half (455) had a primary infection. However, DSS was more prevalent in patients with secondary infections (112/535) than in those with a primary infection (43/455). The median age of patients with primary infections (5 years) was not significantly different (p < 0.05, Wilcoxon rank sum) from those with a secondary infection (6 years). Thirty-nine of the primary infections occurred in children <1 year of age; shock developed in 7 of these children. Of the reminder, 17 had DF, 12 had DHF grade I, and 3 had DHF grade II). Virus (dengue 1) was recovered from the acute-phase serum of three of these seven DSS patients. Two patients <1 year of age had a secondary infection; dengue fever developed in both.

**Figure 1:**
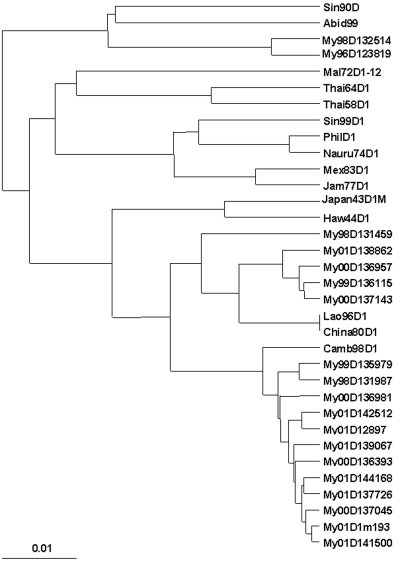
Phylogenetic analysis of the nucleotide sequences of the E protein genes of dengue 1 viruses from Myanmar and of dengue 1 viruses from other localities. Bootstrap values of 100% are shown.

Phylogenetic analyses of the nucleotide sequences of the E protein gene of the only pre-1998 DENV-1 available from Myanmar along with 3 of the 9 isolates from 1998, both 1999 isolates, 5 of the 6 isolates from 2000, and 8 of the 115 isolates from 2001 (including an isolate recovered from a single female *A. aegypti* mosquito [My01D1m193] collected in the home of a dengue patient [My01D141500]) suggested that two new strains of DENV-1 had appeared some time before 1998, i.e., all three clades of Myanmar DENV-1 viruses have 1998 viruses in them (Figure 1). The clade containing the 1996 isolate (My96D123819) may be extinct (no examples have been identified since 1998). There was no apparent segregation of the viruses in the two most recent clades of Myanmar viruses according to the township (suburb) where the patient lived or to the date of onset of symptoms, i.e., viruses from both clades appeared to be co-circulating.

There were 210 nucleotide differences between the sequences of the E protein genes of the My96D123819 and My98D132514 viruses and those of the remaining Myanmar viruses. Forty-six of these resulted in amino acid changes. Amino acid changes at E37 (N-D), E155 (T-S), E161 (I-T), E329 (A-T), E369 (T-E), E442 (A-T), E468 (I-N) and E492 (T-V) distinguished these two viruses from all other Myanmar DENV-1.

The nucleotide sequences of the E genes of the viruses recovered from a patient and a female *A. aegypti* mosquito from the same house varied at three sites. Two of the changes were silent, and the third resulted in a nonconservative amino acid change at E261 from R (in the patient) to H (in the mosquito). Virus from one other patient (My00D136957) had R at this position, but virus from all other patients had the same amino acid as the mosquito at this site.

## Discussion

The dengue outbreak in Myanamar in 2001 occurred at a time not unanticipated from the usual 3- to 4-year cycles of outbreaks in that country (Table 1). Nevertheless, we are unaware of any previous examples of dengue outbreaks, in countries in which all four dengue virus serotypes are circulating, in which a single serotype has risen to the prominence that DENV-1 appears to have reached in Myanmar in 2001.

**Figure 2:**
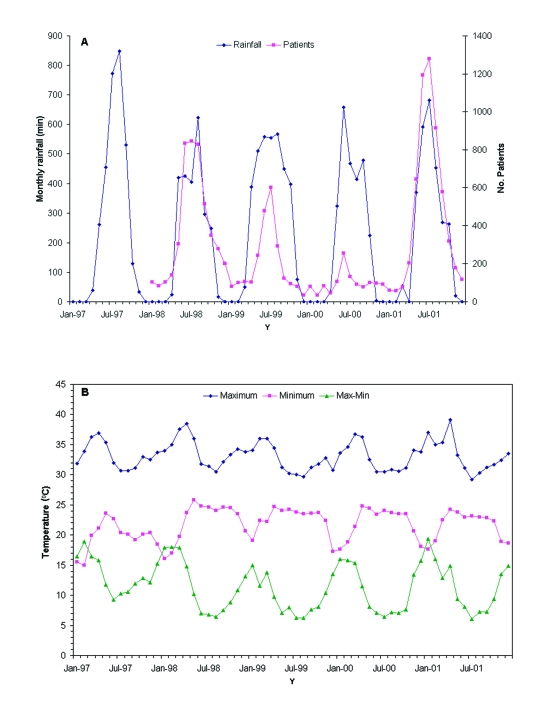
Relationship between weather conditions and the number of cases of dengue in Yangon, 1998–2001. (a) Comparison of the monthly admissions of dengue (dengue fever, dengue hemorrhagic fever, and dengue shock syndrome) patients to the Yangon Children’s Hospital with the total monthly rainfall. (b) The average monthly maximum and minimum temperatures in Yangon from 1997 to 2001 and the difference between these values.

The number of dengue cases in Yangon from 1998 to 2001 was not obviously correlated with the temperature or rainfall (Figure 2) other than the fact that the average temperature in April of the 2 epidemic years (1988, 38.5°C; 2001, 39.1°C) was almost 2°C higher than the highest average in the nonepidemic years. These observations are in broad agreement with those made in Bangkok over much larger periods (*4*,*5*) that weather was not a major factor in determining when dengue outbreaks occurred. However, associations between meterologic conditions and dengue outbreaks could be missed if comparisons are made between aggregated monthly totals (rainfall) or monthly averages (temperature) rather than with daily values.

Why or how the change in the composition of dengue virus populations circulating in Yangon occurred is not clear. If the DENV-1 populations diversified in the 1994–1998 interepidemic period, the changes could have been due to a genetic bottleneck, such as has been proposed to explain the disappearance of ancestral strains and the appearance of new strains of DENV-2 and -3 in Thailand (*16*,*17*). Strong immunologic pressures on dengue virus populations during rapid transmission in outbreaks could give rise to serologic-escape mutant populations of virus (*18*). In the 1998 dengue outbreak in Yangon, in which DENV-1–4 were recovered from patients, all DENV-1 analyzed in this study were recovered in August and September, just after the season had peaked (June 835 DHF/DSS cases; July 845 DHF/DSS cases; August 829 DHF/DSS cases; September, 516 DHF/DSS cases). Six of the eight amino acid changes (see above) that distinguished the two post-1998 DENV-1 lineages from the earlier one occurred in the portion of the E protein above the lipid membrane of the virion (E468 and E492 are in the putative transmembrane anchor region of the E protein [*25*]). The amino acids at E 155 of the pre-1998 lineage of DENV-1 and all 1998 strains of DENV-2–4 were the same (T) and differed from that at this position in the post-1998 DENV-1 lineages (S). At E37 and 442, the pre-1998 lineage DENV-1 isolates shared the same amino acid as 1998 lineages of DENV-2 and -3 and of DENV-2, respectively, and these differed from the amino acids in the corresponding positions of the post-1998 DENV-1 lineages (Table 3). These changes might be taken as evidence of selective pressure imposed on DENV-1 by cross-reactive antibodies produced against the co-circulating DENV-2, -3, or -4. Despite these observations, others have been unable to find any evidence of selection acting on the E protein of DENV-1 in nature (*19*). Furthermore, gaps in the regional virologic record make it difficult to determine whether the post-1998 strains of DENV-1 in Myanmar were introduced or whether they evolved locally.

**Table 3:** Comparison of amino acid changes in dengue 1 virus (DENV-1) E proteins with the sequence of the same region of co-circulating strains of DENV 2–4^a^

Y	Position	Amino acid sequence			
		DENV-1	DENV-2	DENV-3	DENV-4
1998	E37	MAK**N**KPT	MAK**N**KPT	MAK**N**KPT	MAQ**G**KPT
Post-1998		MAK**D**KPT			
1998	E155	GNE**T**TEH	GND**T**GKH	GND**T**QGH	GND**T**SNH
Post-1998		GNE**S**TEH			
1998	E369	IEA**T**PPF	IEA**E**PPF	IEA**E**PPF	IEL**E**PPF
Post-1998		IEA**E**PPF			
1998	E442	IFG**A**AYG	VFG**A**IYG	DFG**S**VGG	VFG**S**VYT
Post-1998		IFG**T**AYG			

^a^Amino acids in bold occur at the positions in the E protein indicated, e.g., E37, E155.

One further difference between the observations of the dengue outbreaks in Yangon from 1984 to 1988 (*7*) and in 2001 was the proportion of dengue patients who had primary infections. From 1984 to 1988, 15% of patients admitted to the Yangon Children’s Hospital had a primary infection (*7*). In 2001, 455 (46%) of 990 virologically or serologically confirmed dengue patients had a primary infection. This increase in the proportion of patients with primary infections may have contributed to the decrease in the proportion of DSS cases (*6*,*7*). Other researchers (*8*) have observed that primary infections with DENV-1 and -3 result in clinical disease more frequently than primary infections with DENV-2 or -4. The data from Myanmar in 2001 are compatible with these observations if the rate of isolation of each dengue virus serotype from patients in Myanmar in 2001 reflected the infection rate with each type in the community at large. The observation of a lower incidence of DSS among patients in 2001, when most infections may have been due to DENV-1, than from 1984 to 1988 (*7*) or in 1998 and 1999 agrees with previous observations that DSS occurs most commonly after infections with DENV-2 in hosts who have had a prior infection with another dengue serotype (*6*,*7*). This report provides further evidence of the ability of dengue viruses to undergo rapid and unpredictable changes in genotype (*16*,*17*) and of the association of such changes with major changes in disease incidence and severity.
